# Improving Muscle Function Through a Multimodal Behavioural Intervention for Knee Osteoarthritis and Obesity: The POMELO Trial

**DOI:** 10.1002/jcsm.70025

**Published:** 2025-07-31

**Authors:** Kristine Godziuk, Mary Forhan, Flavio T. Vieira, Joao F. Mota, Jason Werle, John A. Batsis, Lorenzo M. Donini, Mario Siervo, Carla M. Prado

**Affiliations:** ^1^ Department of Agricultural, Food and Nutritional Science Faculty of Agricultural, Life and Environmental Sciences, University of Alberta Edmonton Canada; ^2^ Department of Physical Therapy and Rehabilitation Science University of California San Francisco USA; ^3^ Department of Occupational Science and Occupational Therapy Temerty Faculty of Medicine, University of Toronto Toronto Canada; ^4^ Department of Pediatrics Faculty of Medicine and Dentistry, University of Alberta Edmonton Canada; ^5^ Faculty of Nutrition Federal University of Goias Goiânia Brazil; ^6^ Section of Orthopaedic Surgery, Department of Surgery University of Calgary Calgary Canada; ^7^ Division of Geriatric Medicine School of Medicine, University of North Carolina at Chapel Hill Chapel Hill USA; ^8^ Department of Nutrition Gillings School of Global Public Health, University of North Carolina at Chapel Hill Chapel Hill USA; ^9^ Experimental Medicine Department Sapienza University Rome Italy; ^10^ School of Population Health, Curtin University Perth Australia

**Keywords:** body composition, exercise, intervention, knee osteoarthritis, multimodal intervention, nutrition intervention, obesity, physical function, sarcopenic obesity

## Abstract

**Background:**

Treatments aimed at improving physical function and body composition, including reducing fat mass (FM) and increasing muscle mass, may benefit individuals with advanced knee osteoarthritis (OA) and obesity. We investigated the feasibility and efficacy of a multimodal behavioural intervention compared to usual care to enhance physical function and muscle mass in this population.

**Methods:**

The POMELO (Prevention Of MusclE Loss in Osteoarthritis) study is a two‐arm pilot randomized controlled trial; NCT05026385. Participants aged 40–75 years, with a BMI ≥ 35 kg/m^2^ and knee OA were randomized 1:1 to either the intervention group (POMELO) or usual care (UC). The 3‐month POMELO intervention incorporated progressive resistance exercise (3 sessions/week), individualized nutrition counselling targeted for OA, and 12 group education sessions on nutrition and arthritis self‐management. The UC group received standard clinical care. After the 3‐month supervised intervention, both groups were followed for 6 months without support. Assessments at baseline, 3 months and 9 months (primary endpoint) included body composition (DXA, measuring FM and appendicular lean soft tissue [ALST]), physical function (chair‐sit‐to‐stands [CSTS], 6‐min walk [6MWT], maximal handgrip strength [HGS]), and health‐related quality of life (Euroqol visual analog scale [EQ‐5D VAS]). Co‐primary outcomes were feasibility (intervention completion ≥ 80% and per‐protocol adherence ≥ 60% [i.e., attendance at 12 education sessions and exercise 3 ×/week]) and acceptability (4‐item Likert‐scale satisfaction survey, and open‐ended questions). Secondary outcomes included changes in physical function and ALST.

**Results:**

Fifty participants were randomized (POMELO = 25, UC = 25), with 32 completing the study (69% female, mean age 64.9 ± 1.2 years, BMI 42.1 ± 1.0 kg/m^2^). The POMELO intervention group had 80% completion and 74% adherence, confirming feasibility. Higher satisfaction rates were observed in POMELO compared to UC (3.5 vs. 2.2, *p* < 0.001) indicating greater acceptability. The POMELO group had improvements in CSTS (mean difference [MD] 3.96, ES 1.2, *p* < 0.001), 6MWT (MD 31.6 m, ES 0.4, *p* = 0.039) and EQ‐5D VAS (MD 7.9 points, ES = 0.4, *p* = 0.01) compared to UC. Both groups experienced FM loss, but only the UC group lost ALST and HGS.

**Conclusion:**

The POMELO intervention, combining personalized nutrition, resistance exercise and self‐management support, was feasible, acceptable and showed greater efficacy than usual care to improve physical function in patients with knee OA and obesity. Our pilot study of this intervention showed potential benefits on body composition and quality of life without focusing on weight reduction. A larger study is needed to confirm these results, as this approach may offer advantages over usual care, potentially leading to better mobility and health outcomes.

## 1 Introduction

Clinical management guidelines for knee osteoarthritis (OA) advise weight loss as a treatment priority for individuals with obesity (defined by body mass index [BMI] ≥ 30 kg/m^2^) to decrease knee joint load, inflammation and pain [1]. However, meta‐analyses highlight the small to modest effect size of weight loss on pain, inflammation and disability in knee OA [2, S1], with low‐quality evidence and no impact on reversing structural damage to cartilage and bone [3]. The potential benefits of weight loss may be primarily in early knee OA [4], with limited effectiveness data and uncertainties of benefit in advanced OA [5]. While some uncertainty may be due to limitations with BMI‐based definitions of obesity [6], weight loss is widely considered a low‐harm recommendation in OA management [S2], including prior to arthroplasty [7].

A balanced assessment of risks and benefits of weight loss for individual patients with OA may be beneficial. Weight loss is known to improve metabolic health in those with abnormal fat mass (FM) or impaired fat tissue function, but there are potential negative consequences on body composition and muscle function to consider. Caloric restriction for weight loss can lead to substantial muscle loss [8]. Excessive caloric restriction in OA without concomitant interventions may contribute to or exacerbate the development of sarcopenic obesity (SO), a clinical condition where low muscle mass and function co‐occur with high FM [9]. SO is associated with negative consequences on patient quality of life, mobility, surgical outcomes and mortality [10]. This condition is highly relevant in individuals with knee OA [11] due to overlapping and accumulating risk factors such as muscle disuse, inflammation and mobility limitations, combined with genetic predisposition, aging and lifestyle factors, including poor diet and inactivity, which together can create an optimal environment for SO development [10]. Up to one in four individuals with obesity and advanced knee OA may have unrecognized SO [11]. While weight loss does not universally result in SO development [12], the prevention of SO should be paramount in individuals at high risk for muscle and function loss (e.g., older individuals with OA).

Concurrent knee OA and obesity can result in a complex interplay of factors that increase the risk for muscle loss, OA disease progression, FM gain and SO development [13]. While the exact direction of associations is not clear, a potential vicious cycle may occur whereby higher FM contributes to increased body mass and mechanical stress on the knee, leading to pain and disuse atrophy, which may worsen joint loading and influence inactivity, reduced energy expenditure and FM gain [13]. Further, fat tissue dysfunction can influence local and systemic inflammation, resulting in worse OA pain and alterations in muscle and joint tissue [14, S3]. As both fat and muscle mass have relevance in structural and symptomatic knee OA [15], strategies aimed at improving body composition—such as reducing FM while preserving or increasing muscle mass—and enhancing physical function and mobility may have greater benefits than solely focusing on absolute weight reduction in patients with advanced OA. However, few studies have explored this strategy, which requires a shift from traditional weight‐loss‐focused interventions to targeted resistance exercise and nutritional strategies to reduce FM while simultaneously improving muscle function, muscle mass and mobility in patients with OA. This approach to prioritize improvements in body composition, including muscle health during weight change could play a crucial role in SO prevention and improving OA management; however, studies are needed in this regard.

To our knowledge, a weight‐neutral multimodal intervention approach has not yet been tested in individuals with both obesity and advanced knee OA. Given this gap, our study evaluated the feasibility, acceptability, and efficacy of a multimodal behavioural intervention designed to improve body composition and physical function compared to usual care in middle‐aged and older adults with knee OA and obesity. We hypothesized that the intervention would be acceptable and would improve muscle function, muscle mass and mobility compared to standard clinical care.

## 2 Methods

### 2.1 Study Design

POMELO (Prevention Of MusclE Loss in Osteoarthritis) was a two‐arm pilot randomized clinical trial (RCT) conducted September 2021–July 2023 (Clinicaltrials.gov: NCT05026385). Ethics approval was received from the University of Alberta in 2021 (Pro00107201), and the study protocol was published in 2022 [16]. The intervention was developed through patient engagement [S4], and decisions on the conduct of the RCT were supported by a patient advisory team with lived experience. Trial details are reported according to CONSORT (Consolidation of Standards of Reporting Trials) guidelines [S5].

### 2.2 Enrollment and Randomization

Recruitment was conducted at a centralized orthopaedic surgery assessment clinic between September 2021 and October 2022. A research assistant invited eligible participants to enroll if they met inclusion criteria. This included BMI ≥ 35 kg/m^**2**^, age 40–75 years, able to communicate in English, no prior arthroplasty or bariatric surgery, and confirmed knee OA based on clinical symptoms, Kellgren–Lawrence (KL) grade ≥ 2 and consulting with the orthopaedic clinic for joint replacement [16]. Randomization was conducted using concealed allocation, whereby an independent statistician generated the allocation sequence, which was blinded to the study team to eliminate potential bias [16]. Consenting patients completed baseline assessments and were subsequently randomized to intervention (POMELO) or usual care (UC) at a 1:1 ratio.

### 2.3 Intervention

The POMELO intervention group received a 3‐month personalized multimodal behavioural health program encompassing three components: (1) progressive resistance exercise, (2) targeted nutrition consult and education and (3) arthritis self‐management support. Details of the intervention are reported in the protocol paper [16]. The intervention components and education were designed to be weight‐neutral (i.e., no emphasis on weight loss, which was communicated to participants) and support improvements in muscle health and OA management for individuals with a higher BMI (i.e., ≥ 35 kg/m^2^). The intervention involved a whole‐body resistance exercise program of 8 exercises personalized and progressed over the 12 weeks with support from a clinical exercise physiologist (CEP). Participants received an initial one‐hour individual consultation with the CEP for exercise instruction, personalized to individual capacity based on their knee OA symptoms and other musculoskeletal conditions. Individuals were asked to complete the exercises 3 ×/week, either independently, supervised in‐person, or via videoconferencing sessions, according to their preference [16]. Exercise equipment was provided for home use, and the CEP conducted biweekly check‐ins with each participant during the intervention period to assist with adaptations and check on exercise progression and adherence. All participants kept a record of exercise completion during the 3‐month intervention and returned this to the study team.

Targeted nutrition recommendations were provided during an initial one‐hour baseline consultation with a registered dietitian (RD), focusing on improved protein quantity, quality and distribution across meals, overall improved diet quality and maintaining an isocaloric diet (i.e., balanced energy intake with expenditure) [16]. Nutrition education recommendations were designed to improve muscle health [17] and reduce OA‐related inflammation [18]. The baseline consultation was followed by weekly one‐hour group discussion sessions held each week for 12 weeks via videoconferencing, alternating between nutrition education, led by the RD and arthritis self‐management, led by an occupational therapist. Self‐management education was included to support behaviour modification regarding exercise and nutrition and improve self‐efficacy for managing OA‐related pain and mobility limitations [19]. Details of the nutrition and self‐management education sessions have been previously published [16]. After the 3‐month supervised intervention, both the POMELO and UC groups were followed for 6 months without support. No information was provided to either group during this period.

### 2.4 Usual Care ([UC] Control)

The UC group followed standard clinical care practices and continued their routine arthritis management under the guidance of the orthopaedic clinic. Since arthritis is a dynamic condition, participants were allowed to make behavioural adjustments to self‐manage their condition. As part of clinical practice, UC participants may have received clinical‐guideline information about OA, including self‐management techniques and advice for exercise, physical therapy or weight management [S6]. UC participants were not restricted in their activities, and they may have independently chosen to exercise or reduce caloric intake based on recommendations for weight reduction from their physician or orthopaedic clinic to meet BMI criteria for arthroplasty [20]. No guidance or advice was provided by the study intervention staff.

### 2.5 Assessments

Study participants completed in‐person assessments at baseline, 3 months and 9 months at the Human Nutrition Research Unit, University of Alberta [16]. Measurements included anthropometrics (e.g., height, weight, BMI, waist and calf circumference) and total and regional body composition (dual‐energy x‐ray absorptiometry, GE Healthcare Lunar iDXA, ENCORE software version 18). Bone mineral content, FM, lean soft tissue (LST) and appendicular LST ([ALST], LST of arms and legs) were collected. ALST was used as a surrogate of muscle mass [21].

Physical function assessments completed at each visit included 30‐s chair sit‐to‐stands (CSTS), the six‐minute walk test (6MWT), and absolute maximal handgrip strength (HGS). Details for each assessment are reported in the protocol [16]. Briefly, CSTS was assessed by the number of completed repetitions moving from seated to full stand in 30‐s; 6MWT was the distance in meters walked in a 6‐min timed interval on an indoor course; HGS was the highest grip strength scored of three attempts in each hand measured with a Jamar hydraulic dynamometer.

Patient‐reported outcomes were collected through online questionnaires prior to each study visit using an electronic research data system (REDCap). Knee OA pain, stiffness and function were assessed with the Western Ontario and McMaster Universities Osteoarthritis Index (WOMAC) [S7], with higher scores indicating worse status. Self‐efficacy for chronic disease management was reported on the Arthritis Self‐Efficacy Scale (ASES) [S8], assessed in three domains (pain, function, and other symptoms, including fatigue, pacing, depression and frustration). Higher scores on ASES indicate greater self‐efficacy. Health‐related quality of life was rated on the Euroqol Foundation (EQ‐5D) [S9] visual analog scale (VAS) from 0 mm (worst health) to 100 mm (best health).

### 2.6 Study Outcomes

#### 2.6.1 Co‐Primary Outcomes

Co‐primary outcomes were the feasibility and acceptability of the POMELO intervention. Feasibility was determined by a priori targets [16] for intervention completion of ≥ 80% and per‐protocol adherence ≥ 60% (i.e., attendance rate at 12 weekly education sessions and completion of resistance exercises 3 ×/week for 12‐weeks), based on prior OA trials [22]. Acceptability was assessed with an online survey (Data S1) assessing satisfaction with the treatment (POMELO or UC) related to improvements in arthritis management, pain, and ability to do home and recreational activities. Each of four questions were scored on a 4‐item Likert scale from *very dissatisfied* (1) to *very satisfied* (4). Additionally, participants were asked to provide free‐text responses to open‐ended survey questions about changes in their health resulting from the treatment randomization. Responses were evaluated using content analysis to identify perceived effectiveness of the intervention or UC as a component of acceptability [23].

#### 2.6.2 Secondary and Exploratory Outcomes

Efficacy outcomes (secondary) were assessed by comparing between‐group changes (POMELO versus UC) in physical function (CSTS and 6MWT) and muscle mass (ALST) from baseline to 3 months and 9 months. Exploratory outcomes included changes in HGS, self‐reported health‐related quality of life (EQ‐5D VAS), self‐efficacy (ASES) and pain and physical function (WOMAC).

#### 2.6.3 Sarcopenic Obesity (SO) Prevention

Changes in individuals meeting diagnostic criteria for SO among study completers were explored over the 9 months to assess implications of the intervention or UC for SO prevention. Diagnostic criteria from the sarcopenic obesity global research initiative (SOGLI) consensus group were used to define SO [9]. Obesity was confirmed at baseline in all study participants based on BMI, waist circumference (above thresholds associated with optimal health outcomes [> 101.2 cm in females, > 103 cm in males] [S10]) and measured FM (high %FM > 40% in females [F], > 30% in males [M] [S11] for White or > 41% F, > 29% M in Black or Asian racial groups [S12]). Low muscle function was identified by low absolute maximal HGS (< 20 kg for females, < 30 kg for males [S13]). Low muscle mass was identified using sex‐specific ALST/weight cut‐offs based on mixed‐ethnicity populations (< 19.4% for females, < 25.7% for males [S14]). Individuals who met all criteria (i.e., low muscle function + low muscle mass + obesity) were identified with SO.

### 2.7 Analyses

The study sample size was determined a priori for feasibility (*n* = 50, 25 per group) with 80% power to detect a low to moderate effect size (ES) in physical function (CSTS and 6MWT) and ALST outcomes accounting for anticipated drop‐out of 16% [16, 24]. Data distribution and homogeneity of variance was checked by Shapiro–Wilk and Levene's test, respectively. Descriptive analyses are reported as mean (standard deviation), median (interquartile range) or frequency (proportion). Baseline comparisons were conducted with Student's t‐test, Mann Whitney U test or Fischer's exact test, as appropriate based on data distribution and outcome. Per‐protocol analyses were conducted to assess the effects of the POMELO intervention compared to UC at 9 months, using analysis of covariance (ANCOVA), controlling for baseline values, with Bonferroni posthoc correction. The ES of between‐group differences in change from baseline to 9 months was estimated using Cohen's d. Mann Whitney U test was used for between‐group comparisons of satisfaction. All testing was two‐tailed and *p*‐values < 0.05 were considered significant. Quantitative analyses were completed using IBM SPSS Statistics v29 (IBM Corp. Armonk, NY).

Preliminary qualitative analyses were conducted on open‐text survey responses regarding acceptability. Two research team members independently coded responses under constructs from a framework of acceptability of healthcare interventions, which includes perceived effectiveness as a construct of acceptability [S15]. Initial themes related to the effectiveness of the POMELO intervention or UC were identified from coded data using deductive content analysis.

## 3 Results

**FIGURE 1:**
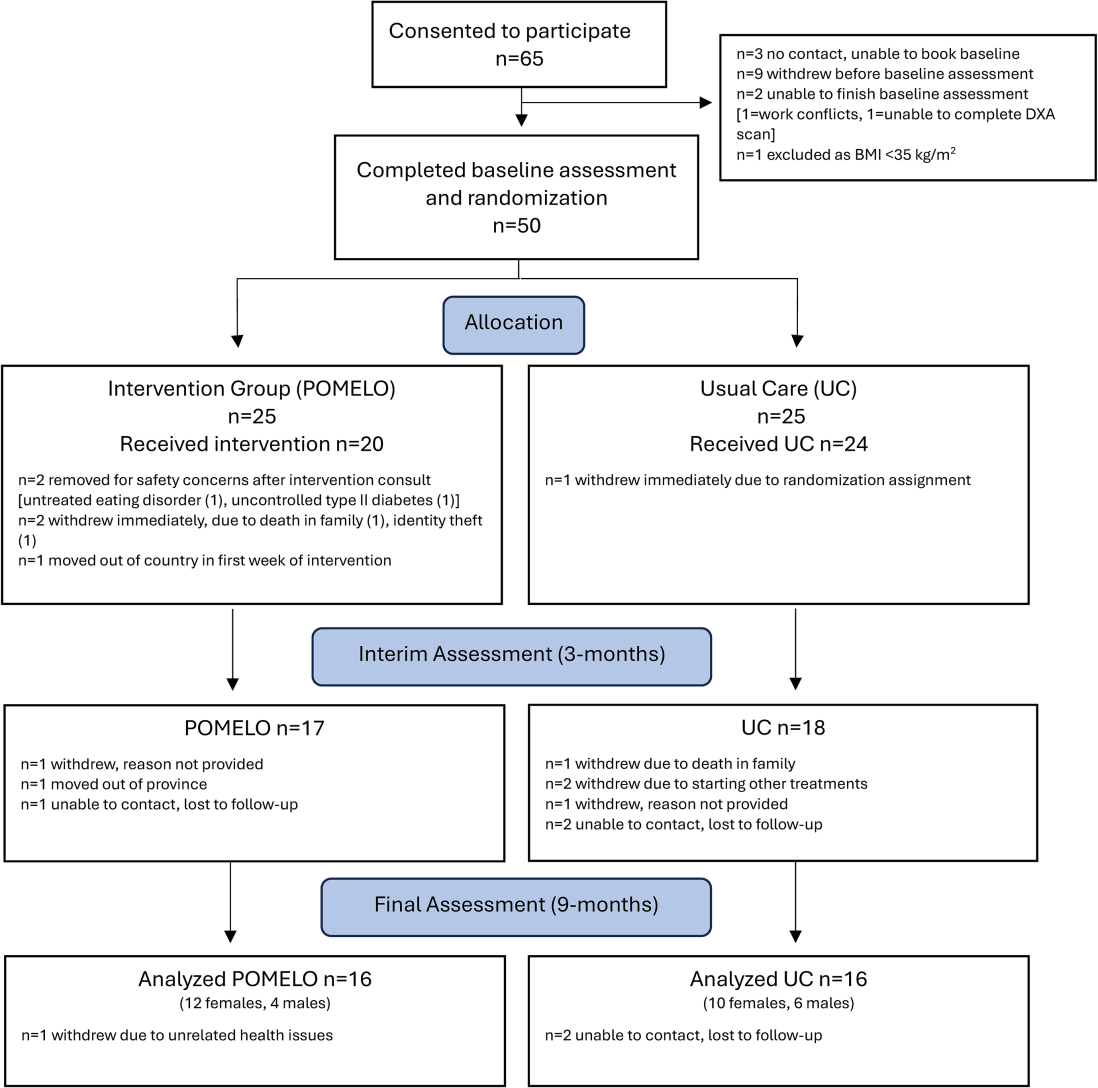
CONSORT flow diagram of study participants with advanced knee osteoarthritis and a body mass index ≥ 35 kg/m^2^. DXA = dual‐energy x‐ray absorptiometry, POMELO = intervention group, UC = usual care group (control).

Sixty‐five adults consented to participate, with 50 completing baseline assessments and subsequently randomized (*n* = 25 POMELO intervention, *n* = 25 UC, Figure 1, with mixed composition of males and females in each group). All participants were verified to have KL grade > 3 based on physician reports. After randomization, the groups had no difference in baseline scores, except for the UC group having a higher BMI, weight, waist circumference and FM (kg) (Table 1). Thirty‐two individuals completed the study (*n* = 16 per group) and were included in per‐protocol analyses. Study completers were 69% female, mean age 64.9 ± 1.2 years, BMI 42.1 ± 1.0 kg/m^2^. There were no differences between study completers and non‐completers (Data S2). Attrition of study participants was high in the first year, aligning with escalations in COVID‐19 public health measures (Data S3).

**TABLE 1:** Description of intervention (POMELO) and control (usual care) groups at baseline (*n* = 50 total).

	Usual care			POMELO		
	Total *n* = 25	Females *n* = 17	Males *n* = 8	Total *n* = 25	Females *n* = 20	Males *n* = 5
**Demographics**						
Females, *n* (%)	17 (68)	—	—	20 (80)	—	—
Age, years	62.2 (7.2)	62.0 (7.1)	62.7 (8.1)	65.4 (6.3)	65.9 (6.3)	63.6 (6.8)
Age category, ≥ age 65 years, *n* (%)	10 (40)	7 (41)	3 (37)	16 (64)	13 (65)	3 (60)
Race or ethnicity, self‐reported, *n* (%)						
White	22 (88)	16 (94)	6 (75)	23 (92)	19 (95)	4 (80)
Indigenous	3 (12)	1 (6)	2 (25)	0 (0)	0 (0)	0 (0)
Black	0 (0)	0 (0)	0 (0)	1 (4)	1 (5)	0 (0)
South Asian	0 (0)	0 (0)	0 (0)	1 (4)	0 (0)	1 (20)
Education > high school, *n* (%)	17 (68)	11 (65)	6 (75)	21 (84)	17 (85)	4 (80)
Bilateral knee osteoarthritis, *n* (%)	21 (84)	15 (88)	6 (75)	18 (72)	18 (90)	0 (0)
Self‐reported severe osteoarthritis, *n* (%)	19 (76)	11 (65)	8 (100)	17 (68)	13 (65)	4 (80)
**Anthropometrics and body composition**						
Weight, kg	120.8 (13.7)^a^	116.5 (12.1)	129.9 (13.1)	108.8 (14.8)^a^	105.7 (12.9)	121.5 (16.8)
Height, meters	1.66 (0.07)	1.63 (0.05)	1.73 (0.05)	1.64 (0.08)	1.60 (0.06)	1.78 (0.04)
Body mass index, kg/m^2^	43.7 (3.9)^a^	44.0 (4.1)	43.3 (3.4)	40.5 (4.8)^a^	41.0 (4.9)	38.3 (4.1)
Waist circumference, cm	130.6 (8.8)^a^	127.8 (7.7)	136.5 (8.4)	120.8 (9.7)^a^	118.8 (8.6)	128.9 (10.8)
Fat mass, kg	60.3 (9.2)^a^	62.8 (7.8)	55.1 (10.2)	53.7 (11.0)^a^	55.1 (11.0)	48.1 (10.2)
Fat mass, %	50.5 (6.1)	54.1 (2.1)	42.7 (4.1)	49.9 (7.0)	52.5 (4.8)	39.5 (3.8)
Fat mass/height^2^, kg/m^2^	8.0 (1.8)	9.0 (1.3)	6.1 (0.9)	7.6 (2.2)	8.3 (1.9)	4.8 (0.9)
Appendicular lean soft tissue (ALST), kg	26.8 (5.2)	23.8 (2.9)	33.0 (2.8)	24.0 (4.9)	21.9 (2.2)	32.4 (3.6)
ALST/height^2^, kg/m^2^	9.6 (1.4)	9.0 (1.0)	11.0 (1.1)	8.8 (1.0)	8.5 (0.7)	10.2 (0.9)
**Physical function, strength and quality of life**						
6‐Minute Walk, meters	352.6 (78.4)	346.2 (78.6)	366.2 (81.5)	382.0 (118.9)	359.1 (114.6)	473.9 (96.5)
Chair sit‐to‐stands, repetitions in 30s	7.9 (3.6)	7.7 (2.7)	8.4 (5.3)	8.1 (3.1)	7.5 (3.0)	10.4 (2.7)
Maximal handgrip strength, kg	34.3 (11.7)	28.3 (6.0)	47.1 (10.6)	31.5 (10.0)	27.2 (4.4)	48.8 (7.0)
EQ‐5D VAS, 0–100	53.5 (18.9)	51.5 (18.7)	57.7 (19.8)	54.9 (20.7)	53.1 (20.3)	62.0 (23.0)

*Note:* Data reported as mean (standard deviation) unless otherwise indicated.

Abbreviation: EQ‐5D VAS = EuroQol visual analog scale.

^a^ Differences between usual care and POMELO groups at baseline.

### 3.1 Feasibility and Acceptability

Co‐primary outcome targets for feasibility and acceptability were met or exceeded (Tables 2 and 3). In participants who initiated the POMELO intervention period, study completion was 80%, and adherence to the intervention protocol was 74% (Table 2). Satisfaction scores at 9 months were higher in POMELO versus UC (median 3.5 [IQR 3.0, 3.94] vs. 2.2 [IQR 1.6, 2.9] on 4‐item Likert scale) (Table 2), and predominant themes of ‘Improving’ and ‘Resilience’ were identified from POMELO participants' open‐text survey responses (Table 3), supporting the acceptability of the intervention. No adverse events were reported to research personnel during the intervention period.

**TABLE 2:** Feasibility and acceptability of POMELO multimodal behavioural intervention in individuals with advanced knee osteoarthritis and a body mass index ≥ 35 kg/m^2^.

	A priori target	Result	Target achieved
**Feasibility**			
Intervention completion^a^	≥ 80%	80%^a^	Yes
Intervention adherence^b^	≥ 60%	74%^b^	Yes
**Acceptability**			
Self‐reported satisfaction at 9‐months (median [IQR] of 4 questions scored on a 4‐item Likert scale)^c^	Greater satisfaction in POMELO vs. usual care group	3.5 (3.0, 3.9) vs 2.2 (1.6, 2.9), *p < 0.001* ^d^	Yes

Abbreviation: IQR = interquartile range.

^a^ In participants who initiated a 12‐week intervention program (16/20 individuals).

^b^ Attendance at 12 weekly group education sessions (median 8, interquartile range 5–10) and completion of resistance exercise 3 times per week over the 12 weeks (median 27.5, interquartile range 2–34.5); calculated as median overall adherence 35.5/48.

^c^ Survey questions included in the Supporting Information.

^d^ Between‐group comparison at study end conducted using Mann–Whitney test.

**TABLE 3:** Perspectives on acceptability of POMELO intervention and usual care, based on thematic analysis of survey responses from participants in each group^a^.

Themes of acceptability of POMELO	Themes of acceptability of usual care
**Improving**	**Worsening**
*‘*'I am stronger, and fitter, have less pain, and can exercise longer. Do not get me wrong, I still have limitations but not near as limiting as before’. P1	‘My knees are much worse. I have to use crutches all the time now’. UC1
‘My knee pain is much reduced in general. I have had good improvements overall in my physical function and feel stronger’. P2	‘Knee pain has gotten more severe, Advil not as effective as it once was’. UC2
‘The program will not regenerate the damaged knee but it has improved my energy levels and my ability to move around’. P3	‘My knee arthritis got worse—so affected my ability to exercise. More difficult to walk. Very unstable outside, have to use a cane’. UC3
**Resilience**	**Uncertainty**
‘I feel I have a more positive attitude and feel more in control. I feel healthier and am more aware of daily movement and activities’. P4	‘I'm trying weight loss which helped somewhat but not significantly. Lost 27 lbs but am gaining back. Kind of got depressed after feeling like there was no hope to ending the pain and succumbed to cravings’. UC4
‘It definitely had a positive influence on me in every area. Mentally, physically and emotionally. I think I have become a little more patient with myself’. P5	‘My life is very unpredictable day to day because I never know what the pain is going to allow me to do’. UC5

*Note:* Example quotes were taken verbatim from survey responses. Details on open‐ended survey questions are included in the Supporting Information.

^a^ Content analysis of open text survey answers, exploring perceptions on the effectiveness of the intervention or usual care as a component under a framework of acceptability of healthcare interventions.

### 3.2 Efficacy

**FIGURE 2:**
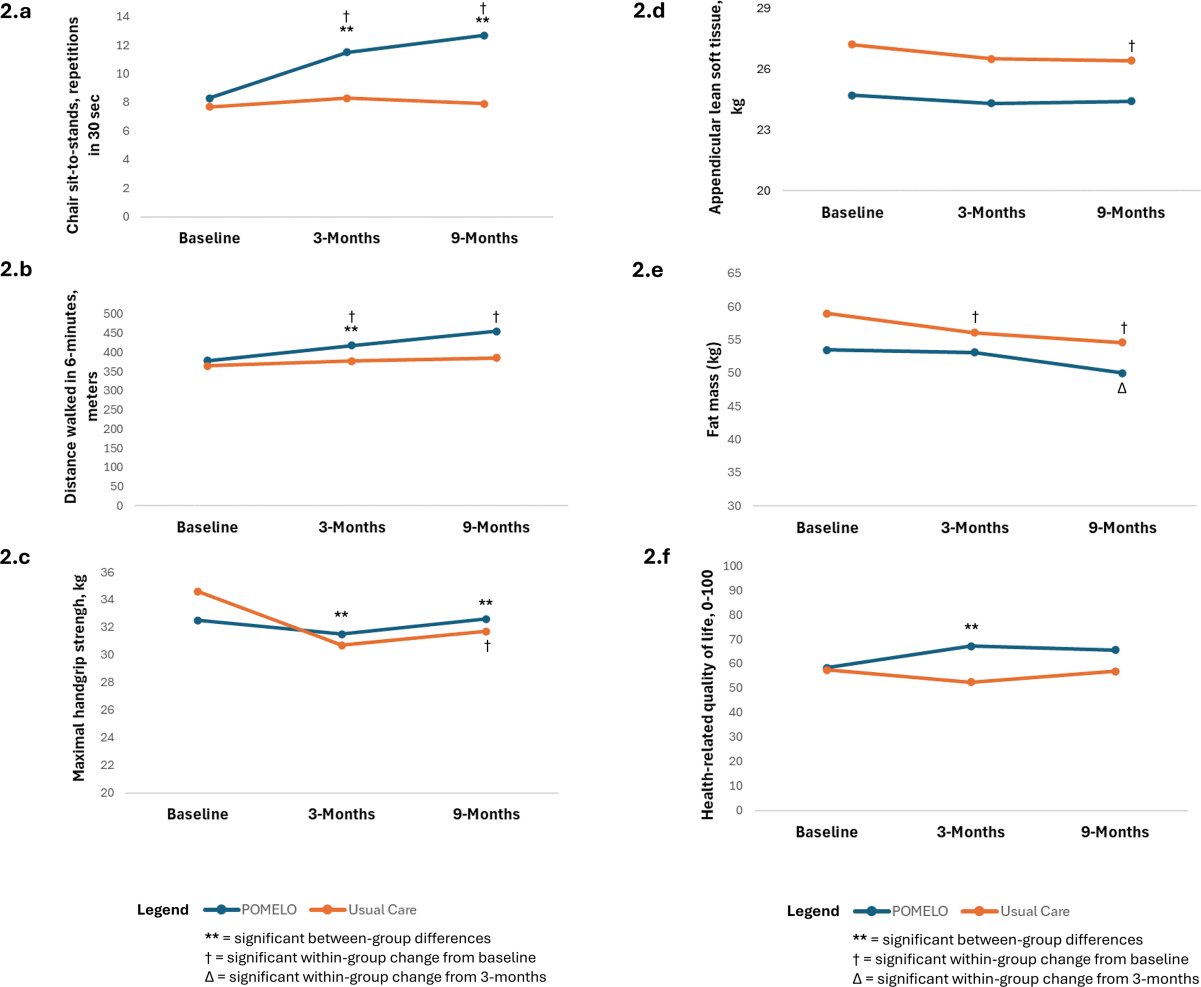
Mean changes in secondary and select exploratory outcomes (2.a‐c, physical function and strength, 2.d. appendicular lean soft tissue, 2.e. fat mass and 2.f., health‐related quality of life) in the POMELO intervention and usual care groups after the 9‐month study period.

The POMELO group had superior improvements in physical function compared to UC over 9 months (CSTS mean difference +3.96 repetitions, ES = 1.2, *p* < 0.001; 6MWT mean difference +31.6 m, ES = 0.4, *p* = 0.039, Figure 2, Table 4). The POMELO group had improved quality of life (EQ‐5D VAS mean difference 7.9 points, ES = 0.4, *p* = 0.010), and self‐efficacy for managing pain (ASES pain mean difference 0.2, ES = 0.1, *p* = 0.015), compared to UC. The UC group had a decline in maximal HGS compared to POMELO (HGS mean difference −3.0 kg, ES = 1.0, *p* = 0.026, Figure 2, Table 4). We also examined HGS normalized to body weight, and the results did not change (HGS/weight mean difference 0.02 kg/kg, ES 0.8, *p* = 0.011). ALST decreased in the UC group after 9 months, with a trend toward a between‐group difference (mean difference −0.5 kg, ES = 0.4, *p* = 0.08, Figure 2, Table 4). Both groups lost absolute FM, and only the UC had within‐group changes in body weight, BMI and %FM. There were no differences between groups in WOMAC scores, data not shown.

**TABLE 4:** Effects of POMELO intervention in comparison to usual care on secondary and exploratory outcomes using per protocol analysis.

	Usual care *n* = 16			POMELO *n* = 16			Between group
	Baseline	3‐Months post	9‐Months post	Baseline	3‐Months post	9‐Months post	*p*
**Secondary outcomes**							
Chair sit‐to‐stands, repetitions in 30s	7.7 ± 4.2	8.3 ± 3.3^a^	7.9 ± 2.1^a^	8.3 ± 3.4	11.5 ± 2.6^a^, ^b^	12.7 ± 3.6^a^, ^b^	*< 0.001*
6‐Minute walk, meters	364.6 ± 81.2	377.0 ± 80.0^a^	385.4 ± 69.3	378.3 ± 134.5	417.5 ± 101.1^a^, ^b^	454.4 ± 114.4^b^	*0.039*
Appendicular lean soft tissue (ALST), kg	27.2 ± 5.3	26.5 ± 4.8	26.4 ± 5.6^b^	24.7 ± 5.6	24.3 ± 5.8	24.4 ± 5.8	*0.08*
**Exploratory outcomes**							
Body mass index (BMI), kg/m^2^	43.4 ± 3.9	42.4 ± 3.9^b^	41.8 ± 4.0	40.8 ± 5.5	40.6 ± 5.5	39.0 ± 5.0	*0.782*
Body weight, kg	120.3 ± 14.7	116.8 ± 14.4^b^	116.1 ± 14.2	109.9 ± 16.7	108.2 ± 17.6	105.5 ± 17.9	*0.832*
Waist circumference, cm	130.2 ± 10.4^d^	129.9 ± 11.9	129.4 ± 10.3	122.3 ± 10.5^d^	119.5 ± 12.2	119.2 ± 11.2	*0.108*
Fat mass, kg	59.0 ± 10.1	56.1 ± 10.4^b^	54.6 ± 10.3^b^	53.5 ± 12.5	53.1 ± 12.8	50.0 ± 11.4^c^	*0.395*
Fat mass, %	49.6 ± 6.1	48.8 ± 6.5^b^	48.0 ± 6.8^b^	49.2 ± 7.9	49.7 ± 7.2	47.8 ± 7.3	*0.297*
Fat mass/height^2^, kg/m^2^	21.3 ± 3.7	20.4 ± 3.6^b^	19.7 ± 3.8^b^	20.1 ± 5.5	20.1 ± 5.3	18.3 ± 4.6^b^	*0.738*
ALST/height^2^, kg/m^2^	9.8 ± 1.5	9.6 ± 1.5	9.5 ± 1.7^b^	9.0 ± 1.1	9.0 ± 1.2	8.9 ± 1.3	*0.276*
Maximal handgrip strength, kg	34.6 ± 10.9	30.7 ± 7.0^a^	31.7 ± 9.6^a^, ^b^	32.5 ± 12.0	31.5 ± 10.1^a^	32.6 ± 12.6^a^	*0.026*
Quality of Life (EQ‐5D VAS), scored 0–100	57.5 ± 20.8	52.5 ± 20.1^a^	56.9 ± 19.3	58.4 ± 19.4	67.2 ± 16.1^a^	65.7 ± 18.2	*0.01*
Arthritis self‐efficacy scale (ASES)							
Pain, scored 1–10	5.0 ± 2.1	4.4 ± 1.7^a^	5.1 ± 1.9	5.7 ± 1.8	6.3 ± 1.8^a^	6.0 ± 2.1	*0.015*
Function, scored 1–10	7.0 ± 1.2^d^	7.1 ± 2.0	7.4 ± 1.5	7.9 ± 1.0^d^	8.2 ± 2.1	8.2 ± 1.3	*0.522*
Other symptoms, scored 1–10	5.1 ± 1.5^d^	5.3 ± 1.7	6.1 ± 1.7	7.0 ± 1.5^d^	7.0 ± 1.9	7.3 ± 1.5	*0.355*

*Note:* Mean values ± standard deviation unless otherwise indicated.

Abbreviations: ALST = appendicular lean soft tissue, EQ‐5D VAS = EuroQol visual analog scale.

^a^ Significant between‐group differences using analysis of covariance controlling for baseline in subjects with complete data at all timepoints.

^b^ Significant within‐group change from baseline.

^c^ Significant within‐group change from 3 months.

^d^ Differences between groups at baseline.

### 3.3 SO Status

Post hoc analyses identified changes in individuals meeting SO diagnostic criteria between baseline and 9 months (Data S4). One POMELO participant resolved their baseline low muscle function, with 2 UC participants experiencing a worsening of muscle function over the 9 months (i.e., maximal HGS). One UC participant (6.2%) developed SO during the 9‐month study due to progression to both low muscle function and low muscle mass. No POMELO group participants developed SO over the 9 months.

## 4 Discussion

This 12‐week multimodal behavioural intervention (POMELO), which included personalized resistance exercise, nutrition education and self‐management support, proved feasible, acceptable and more effective than usual care (UC) in improving physical function and quality of life, and maintaining strength over 9 months in patients with advanced knee OA and obesity. Additionally, only the POMELO group was able to maintain muscle mass. The intervention also enhanced patient self‐efficacy for pain management and showed potential to improve body composition without emphasizing caloric restriction or weight loss. Considering that this population is at high risk for strength loss, persistent functional impairments and the development of SO [25], our findings suggest that multimodal interventions like POMELO could be effective strategies to preserve muscle function (and potentially muscle mass) in the context of knee OA, a highly disabling condition. To our knowledge, this is the first study examining an intervention designed to prevent muscle and strength loss in patients with advanced knee OA and obesity.

Our data highlight the potential risk of SO onset or progression in individuals with advanced knee OA [25]. Notably, the UC group experienced muscle mass and strength losses during the 9‐month study period, with one individual developing SO. SO prevention deserves more attention in the OA field due to overlapping risk factors such as inactivity, inflammation and aging, which contribute to muscle and strength losses [26]. Usual care OA management strategies are unlikely to effectively counteract these declines in this patient group as they predominantly focus on patient weight loss and low‐intensity mobility exercises in preparation for surgery. There is a critical need to improve the identification of at‐risk individuals, and deliver targeted countermeasures to preserve patient physical functioning and muscle mass [27].

The POMELO intervention had clinically relevant positive effects on physical function (i.e., CSTS and 6MWT) based on published data of meaningful change in OA [28], with moderate to large effect sizes, adding to existing evidence supporting progressive resistance exercise to improve muscle strength and physical function in older adults with OA [29]. Our findings demonstrate that a whole‐body progressive resistance exercise program is both acceptable and reasonable to complete from the perspective of individuals with a higher body weight (i.e., BMI ≥ 35 kg/m^2^) and advanced knee OA. Tailoring exercise in this patient population to adjust technique, type and pacing (e.g., rest time between sets or exercises, and order of completion) for OA‐related mobility limitations or psychosocial barriers is particularly beneficial [S16]. While our focus was on resistance exercise, aerobic exercise or a combination of aerobic and resistance exercise could offer additional benefits, such as reducing visceral and intramuscular fat, promoting mitochondrial synthesis and lowering inflammatory biomarkers [27]. Further research will clarify what exercise prescription combinations are ideal for individuals who have knee OA concurrent with different characteristics or clinical conditions (e.g., metabolic syndrome, SO, pain phenotypes), and tailored to specific goals (i.e., prevention or treatment). Existing data suggest that a personalized or precision medicine approach will be critical in addressing the growing relevance of SO across diverse clinical populations [10].

Enhanced protein intake is known to confer benefits to body composition and help prevent age‐related muscle loss, with greater benefits when applied in combination with resistance exercise [30]. The personalized nutrition prescription and counselling component included in the POMELO intervention (i.e., recommendations for improved protein quantity, quality and distribution across meals) [S17] may have contributed to the preservation of muscle mass in this sample of older adults with mobility impairments. Optimized nutritional status can support muscle health through multiple mechanisms, including adequate energy intake and macronutrients to support muscle anabolism and anti‐catabolic and ‐inflammatory effects [10]. Improvements in diet quality may extend to reductions in systemic inflammation and improvements in joint health [31]. Adequate protein intake supports muscle protein synthesis, which is crucial for preserving muscle mass, especially in older adults with knee OA. Additionally, a diet rich in ‘muscle‐building’ nutrients can also benefit muscle mass and function [32].

The observed trend toward greater muscle mass preservation in the POMELO group (i.e., maintained ALST compared to loss of 0.5 kg of ALST in UC) suggests that our study may have been underpowered to fully explore this effect, or that short‐term interventions may be insufficient for substantial muscle gains in these patients, highlighting the need for longer, sustained efforts. The POMELO intervention group retained its muscle mass while the UC group had declined, suggesting the multimodal intervention was sufficient to trigger the anabolic potential of participants. Villareal et al. [33] conducted a 1‐year intervention study in adults over age 65, with mean gains in LST of 1.6 kg reported after 1 year. Other studies in adults > 60 years have reported improvements in skeletal muscle mass of 0.9 kg after 6 months of resistance exercise [34]. Combined exercise and nutrition intervention studies aiming to improve muscle mass in older adults may require a longer intervention period to have a positive effect, due to the attenuation of muscle protein synthesis response with aging [35]. As a result, older adults have a double burden of difficulty with gaining muscle mass combined with a high risk for loss of muscle mass. OA contributes additionally to this burden through both inflammation and potential muscle disuse associated with joint pain, further amplifying the risk for muscle atrophy and strength reduction [36]. Taken together, this highlights the critical need to adopt muscle loss prevention strategies in this clinical population.

Notably, the inclusion of chronic disease self‐management education as a third component in the POMELO intervention may have contributed to improvements in quality of life and self‐efficacy compared to UC. Engaging and counselling patients in effective self‐management strategies and progressive behavioural health changes can enhance their ability to adapt and cope with painful musculoskeletal conditions [S18]. Enhanced self‐efficacy can positively influence patient mindset and resilience in managing OA‐related pain, contributing to improved mobility performance and quality of life [37].

Traditionally, interventions for individuals with obesity and OA have primarily focused on caloric restriction and exercise to promote weight loss and symptom relief [S19]. The long‐term impact of intentional weight loss on muscle and bone health, compared to weight‐neutral exercise and nutrition interventions, should be carefully considered in the management of advanced knee OA management [38]. Unsupervised caloric restriction can increase the risk for malnutrition, muscle and bone loss, which may lead to increased complications during arthroplasty and compromised long‐term implant stability [S20]. There are substantial knowledge gaps in this area, highlighting the need to explore the broader benefits of improved OA care, not only for the patient, but also for the health care system. Precision management for individuals with obesity and knee OA has the potential to deliver substantial patient‐level benefits, such as improved body composition, physical function and health‐related quality of life, as well as health‐system benefits, including reduced uptake or postponement of joint replacement, fewer post‐surgical complications and enhanced implant stability and longevity, which could prevent the need for re‐operations. This area of research requires further attention.

Current literature has yet to establish the optimal interventional approaches for SO prevention versus treatment [39], which may differ across clinical populations. In OA management, effective SO prevention strategies may need to address multiple factors, including preventing weight gain (i.e., in increased FM) to reduce further joint loading, improving body composition (e.g., reducing FM while increasing or preserving muscle mass to conserve muscle forces at the joint [15]), and enhancing patient function, mobility and quality of life [S21]. Obesity treatments, such as glucagon‐like peptide‐1 receptor agonist therapies are of growing interest in OA management [S22]. However, there is uncertainty about the potential for SO development or worsening due to the significant muscle loss associated with these medications [12]. Further research is needed in older adults with OA to assess the implications and therapeutic potential of these medications when combined with resistance exercise and nutrition strategies to improve body composition and clinical outcomes. If medication use is associated with an overall reduction in food intake rather than food preferences [40], then a personalized approach such as the POMELO intervention may have benefits to ensure adequate nutritional intake when implemented with medical interventions.

This study has several strengths. Our patient cohort with a BMI ≥ 35 kg/m^2^ is an under‐represented group in OA and SO intervention studies [16]. Engagement from a patient‐advisory team throughout the study helped to enhance participant enrollment and adherence by addressing patient needs in the design. Furthermore, together with our patient partners, we developed two animated videos to facilitate the knowledge translation of our findings (available at https://www.youtube.com/watch?v=ueDDD01Vr‐8&t=5s). The intervention tested in this study has strong potential to be scalable and cost‐efficient, as individual consultations with the CEP and RD were only required at intervention onset and the remainder of the support could be provided remotely. Cost‐effectiveness modelling is needed in a large study.

This study has some limitations. Dietary adherence was not measured. We used an indirect surrogate measure of muscle mass (i.e., DXA‐assessed ALST), so some caution is needed in the extrapolation of findings [21]. There is potential that baseline differences in BMI and FM between the intervention and usual care groups may have influenced physical function, as body size and obesity severity can impact the results of functional tests [S23]. This pilot used UC as the control, and some benefits may be accrued through attention from intervention delivery. We did not restrict the UC group in the therapies that they used, and the uptake of clinical standard care may have differed among UC participants. Due to higher initial attrition, potentially linked to COVID‐19 public health restrictions (Data S3), our sample size may have limited the ability of some findings to reach statistical significance. Our sample size was based on anticipated 16% dropout, so with 20% dropout, some of our analyses may be underpowered. Nevertheless, these pilot results confirm the feasibility and acceptability of this multimodal intervention and provide preliminary evidence of its efficacy, guiding future studies with larger sample sizes. As our intervention involved multiple elements (i.e., resistance exercise + nutrition strategies + self‐management support), we are unable to discern the individual impacts of each treatment component. Nonetheless, our findings support the potential value of precision multimodal intervention delivery for patients with advanced‐stage OA.

## 5 Conclusion

The POMELO intervention, which included progressive resistance exercise, targeted nutrition education and self‐management support, was feasible, acceptable and more effective than UC in improving physical function and quality of life in patients with advanced knee OA and a BMI ≥ 35 kg/m^2^. It shows promise as a strategy to improve muscle function, reduce disability and prevent SO in this high‐risk population. A larger study is needed to confirm these results.

## Ethics Statement

Provided by the Health Research Ethics Board at the University of Alberta, Edmonton, Alberta, Pro00107201, on March 31, 2021.

## Conflicts of Interest

All authors declare no competing interests that would create a conflict of interest in connection with this manuscript. C.M.P. has received honoraria and/or paid consultancy from Abbott Nutrition, Nutricia, Nestle Health Science, Pfizer, AMRA Medical, Novo Nordisk and funding from Almased for research not related to this study.

## Supporting information




**Data S1‐S4.** Supplementary Information.

## Data Availability

The data can be made available on request pending written application and approval.
