# Supplementary material for: Improving Muscle Function Through a Multimodal Behavioural Intervention for Knee Osteoarthritis and Obesity: The POMELO Trial
Source: J Cachexia Sarcopenia Muscle. 2025 Jul 31;16(4):e70025. doi: 10.1002/jcsm.70025 (PMC12314308; doi:10.1002/jcsm.70025)
Supplement: Supplementary file 1 — Data S1‐S4. Supplementary Information. [file JCSM-16-e70025-s001.docx]

**Title:** Improving muscle function through a multimodal behavioral intervention for knee osteoarthritis and obesity: the POMELO trial

**Journal:** Journal of Cachexia, Sarcopenia and Muscle

**Authors:** K. Godziuk, M. Forhan, F.T. Vieira, J.F. Mota, J. Werle, J.A. Batsis, L.M. Donini, M. Siervo, C.M. Prado

**Supplemental References Cited in Manuscript**

S1. Lim YZ, Wong J, Hussain SM, Estee MM, Zolio L, Page MJ, et al. Recommendations for weight management in osteoarthritis: A systematic review of clinical practice guidelines. Osteoarthr Cartil Open. 2022;4(4). doi:10.1016/j.ocarto.2022.100298

S2. Bowden JL, Hunter DJ, Mills K, Allen K, Bennell K, Briggs AM, et al. The OARSI Joint Effort Initiative: Priorities for osteoarthritis management program implementation and research 2024–2028. Osteoarthr Cartil Open. 2023;5(4):100408. doi:10.1016/j.ocarto.2023.100408

S3. Chang J, Liao Z, Lu M, Meng T, Han W, Ding C. Systemic and local adipose tissue in knee osteoarthritis. Osteoarthritis Cartilage. 2018;26:864-871. doi:10.1016/j.joca.2018.03.004

S4. Godziuk K, Prado CM, Forhan M. Patient engagement in the design of an intervention to prevent muscle loss in individuals with knee osteoarthritis and a body mass index (BMI) ≥ 35. Musculoskeletal Care. 2021. doi:10.1002/msc.1613

S5. Eldridge SM, Chan CL, Campbell MJ, Bond CM, Hopewell S, Thabane L, et al. CONSORT 2010 statement: Extension to randomised pilot and feasibility trials. The BMJ. 2016;355. doi:10.1136/bmj.i5239

S6. Mazzei DR, Whittaker JL, Kania-Richmond A, Faris P, Wasylak T, Robert J, et al. Do people with knee osteoarthritis use guideline-consistent treatments after an orthopaedic surgeon recommends nonsurgical care? A cross-sectional survey with long-term follow-up. Osteoarthr Cartil Open. 2022;4(2). doi:10.1016/j.ocarto.2022.100256

S7. Western Ontario and McMaster Universities Osteoarthritis Index (WOMAC). American College of Rheumatology. http://www.rheumatology.org/I-Am-A/Rheumatologist/Research/Clinician-Researchers/Western-Ontario-McMaster-Universities-Osteoarthritis-Index-WOMAC. Published 2015. Accessed August 23, 2016.

S8. Lorig K, Chastain RL, Ung E, Shoor S. Development and evaluation of a scale to measure perceived self-efficacy in people with arthritis. Arthritis & Rheumatology. 1989;32(1).

S9. Janssen M, Pickard A, Golicki D, Gudex C, Niewada M, Scalone L, et al. Measurement properties of the EQ-5D-5L compared to the EQ-5D-3L across eight patient groups: a multi-country study. Quality of Life Research. 2013;22:1717-1727. doi:10.1007/s11136-012-0322-4

S10. Wang Z, Ma J, Si D. Optimal cut-off values and population means of waist circumference in different populations. Nutr Res Rev. 2010;23(2):191-199. doi:10.1017/S0954422410000120

S11. Woolcott OO, Bergman RN. Defining cutoffs to diagnose obesity using the relative fat mass (RFM): Association with mortality in NHANES 1999–2014. Int J Obes. 2020;44(6):1301-1310. doi:10.1038/s41366-019-0516-8

S12. Gallagher D, Heymsfield SB, Heo M, Jebb SA, Murgatroyd PR, Sakamoto Y. Healthy percentage body fat ranges : an approach for developing guidelines based on body mass index. Am J Clin Nutr. 2000;72:694-701. doi:10.3945/ajcn.2009.28141.

S13. Studenski SA, Peters KW, Alley DE, Cawthon PM, McLean RR, Harris TB, et al. The FNIH sarcopenia project: Rationale, study description, conference recommendations, and final estimates. Journals of Gerontology - Series A Biological Sciences and Medical Sciences. 2014;69 A(5):547-558. doi:10.1093/gerona/glu010

S14. Levine ME, Crimmins EM. The impact of insulin resistance and inflammation on the association between sarcopenic obesity and physical functioning. Obesity. 2012;20(10):2101-2106. doi:10.1038/oby.2012.20.

S15. Sekhon M, Cartwright M, Francis JJ. Acceptability of healthcare interventions: An overview of reviews and development of a theoretical framework. BMC Health Serv Res. 2017;17(1). doi:10.1186/s12913-017-2031-8

S16. Bennell KL, Dobson F, Hinman RS. Exercise in osteoarthritis: Moving from prescription to adherence. Best Pract Res Clin Rheumatol. 2014;28(1):93-117. doi:10.1016/j.berh.2014.01.009

S17. Arentson-Lantz EJ, Layman DK, Leidy HJ, Campbell WW, Phillips SM. Important Concepts in Protein Nutrition, Aging, and Skeletal Muscle: Honoring Dr. Douglas Paddon-Jones (1969-2021) by Highlighting His Research Contributrions. J Nutr. January 2023. doi:10.1016/j.tjnut.2023.01.011

S18. Hutting N, Caneiro JP, Ong’wen OM, Miciak M, Roberts L. Person-centered care for musculoskeletal pain: Putting principles into practice. Musculoskelet Sci Pract. 2022;62. doi:10.1016/j.msksp.2022.102663

S19. Messier SP, Loeser RF, Miller GD, Morgan TM, Rejeski WJ, Sevick MA, et al. Exercise and Dietary Weight Loss in Overweight and Obese Older Adults With Knee Osteoarthritis: The Arthritis, Diet and Activity Promotion Trial. Arthritis Rheum. 2004;50(5):1501-1510. doi:10.1002/art.20256

S20. Zink TM, Kent SE, Choudhary AN, Kavolus JJ. Nutrition in Surgery: An Orthopaedic Perspective. Journal of Bone and Joint Surgery. 2023;105:1897-1906. doi:10.2106/jbjs.23.00259

S21. Schoufour JD, Tieland M, Barazzoni R, Ben Allouch S, Bie J van der, Boirie Y, et al. The Relevance of Diet, Physical Activity, Exercise, and Persuasive Technology in the Prevention and Treatment of Sarcopenic Obesity in Older Adults. Front Nutr. 2021;8. doi:10.3389/fnut.2021.661449

S22. Magruder ML, Jacofsky D, Springer B, Scuderi GR, Hameed D, Mont MA. Semaglutide and Other GLP-1 Agonists: A Boon for the Arthroplasty Industry? Journal of Arthroplasty. 2024;39(2):277-282. doi:10.1016/j.arth.2023.12.014

S23. Donini LM, Poggiogalle E, Mosca V, Pinto A, Brunani A, Capodaglio P. Disability Affects the 6-Minute Walking Distance in Obese Subjects (BMI>40 kg/m2). PLoS One. 2013;8(10). doi:10.1371/journal.pone.0075491

**Additional Supporting References Not Cited Within Manuscript**

Cappellari GG, Guillet C, Poggiogalle E, Ballesteros Pomar MD, Batsis JA, Boirie Y, et al. Sarcopenic obesity research perspectives outlined by the sarcopenic obesity global leadership initiative (SOGLI) – Proceedings from the SOGLI consortium meeting in rome November 2022. Clinical Nutrition. 2023;42(5):687-699. doi:10.1016/j.clnu.2023.02.018

Nutter S, Eggerichs LA, Nagpal TS, Ramos Salas X, Chin Chea C, Saiful S, et al. Changing the global obesity narrative to recognize and reduce weight stigma: A position statement from the World Obesity Federation. Obesity Reviews. 2023;25(1):e13642. doi:10.1111/obr.13642

Thijssen E, van Caam A, van der Kraan PM. Obesity and osteoarthritis, more than just wear and tear: pivotal roles for inflamed adipose tissue and dyslipidaemia in obesity-induced osteoarthritis. Rheumatology. 2015;54(4):588-600. doi:10.1093/rheumatology/keu464

Dondero K, Friedman B, Rekant J, Landers‐Ramos R, Addison O. The effects of myosteatosis on skeletal muscle function in older adults. Physiol Rep. 2024;12(9). doi:10.14814/phy2.16042

Manning KM, Hall KS, Sloane R, Magistro D, Rabaglietti E, Lee CC, et al. Longitudinal analysis of physical function in older adults: The effects of physical inactivity and exercise training. Aging Cell. 2024;23(1). doi:10.1111/acel.13987

Bowden JL, Hunter DJ, Deveza LA, Duong V, Dziedzic KS, Allen KD, et al. Core and adjunctive interventions for osteoarthritis: efficacy and models for implementation. Nat Rev Rheumatol. 2020. doi:10.1038/s41584-020-0447-8

Hutting N, Caneiro JP, Ong’wen OM, Miciak M, Roberts L. Person-centered care for musculoskeletal pain: Putting principles into practice. Musculoskelet Sci Pract. 2022;62. doi:10.1016/j.msksp.2022.102663

Miller GD, Nicklas BJ, Davis C, Loeser RF, Lenchik L, Messier SP. Intensive Weight Loss Program Improves Physical Function in Older Obese Adults with Knee Osteoarthritis. Obesity. 2006;14(7):1219-1230. doi:10.1038/oby.2006.139

Miller GD, Nicklas BJ, Loeser RF. Inflammatory biomarkers and physical function in older, obese adults with knee pain and self-reported osteoarthritis after intensive weight-loss therapy. J Am Geriatr Soc. 2008;56(4):644-651. doi:10.1111/j.1532-5415.2007.01636.x

Miller GD, Jenks MZ, Vendela M, Norris JL, Muday GK. Influence of weight loss, body composition, and lifestyle behaviors on plasma adipokines: A randomized weight loss trial in older men and women with symptomatic knee osteoarthritis. J Obes. 2012;2012. doi:10.1155/2012/708505

Wang X, Miller GD, Messier SP, Nicklas BJ. Knee strength maintained despite loss of lean body mass during weight loss in older obese adults with knee osteoarthritis. J Gerontol A Biol Sci Med Sci. 2007;62(8):866-871.

Beavers KM, Beavers DP, Nesbit BA, Ambrosius WT, Marsh AP, Nicklas BJ, et al. Effect of an 18-month physical activity and weight loss intervention on body composition in overweight and obese older adults. Obesity. 2014;22(2):325-331. doi:10.1002/oby.20607

Beavers DP, Beavers KM, Loeser RF, Walton NR, Lyles MF, Nicklas BJ, et al. The independent and combined effects of intensive weight loss and exercise training on bone mineral density in overweight and obese older adults with osteoarthritis. Osteoarthritis Cartilage. 2014;22(6):726-733. doi:10.1016/j.joca.2014.04.002

Chu IJH, Lim AYT, Ng CLW. Effects of meaningful weight loss beyond symptomatic relief in adults with knee osteoarthritis and obesity: a systematic review and meta-analysis. Obesity Reviews. 2018;19(11):1597-1607. doi:10.1111/obr.12726

**Supplemental 1.**

**Satisfaction Survey**

The satisfaction survey was administered electronically to all study participants remotely before the interim (3-month) and final (9-month) assessment visits. Each of the following questions were scored on a 4-item Likert scale. Respondents were able to select one response for each of the questions using a single-item checkbox. Response options were: 4 (very satisfied), 3 (somewhat satisfied), 2 (somewhat dissatisfied), or 1 (very dissatisfied). The wording in the brackets about group assignment was specific to either the intervention or usual care group i.e. for the intervention group, it specified “the POMELO intervention”, and for the control group, it specified “usual care for knee osteoarthritis”.

***Participant Satisfaction Scale***

***Please answer the following questions about your satisfaction with the results of*** *[randomized group assignment]****.***

1. *How satisfied are you with [randomized group assignment] for management of your knee arthritis?*
2. *How satisfied are you with [randomized group assignment] for improving your pain?*
3. *How satisfied are you with [randomized group assignment] for improving your ability to do home or yard work?*
4. *How satisfied are you with [randomized group assignment] for improving your ability to do recreational activities?*

***Open ended questions***

Additional detailed perspectives from study participants were requested through a series of open-ended questions. These questions were administered electronically after the participant satisfaction scale questions. Participants could type free-text answers into an open-text box with no character limits.

***We would like to hear about your personal experiences and perspective on*** *[randomized group assignment]****. Please answer the questions below:***

1. *Can you tell us about your overall experience with [randomized group assignment]?*
2. *Do you feel that [randomized group assignment] has influenced your knee arthritis symptoms, physical function, or overall health? Please explain.*
3. *Have you experienced any other positive or negative outcomes from [randomized group assignment]? Please describe.*
4. *What would you change about [randomized group assignment], if anything?*
5. *Is there anything else you would like to share or comment on?*

**Supplemental 2.** Brief description of individuals who did and did not complete study

|  | **Completed Study**  **n=32** | **Did Not Complete Study**  **n=18** | **p-value** |
| --- | --- | --- | --- |
| Age, years | 64.9 | 61.7 | 0.1 |
| Sex, female, n (%) | 22 (69) | 15 (83) | 0.33 |
| Body mass index, kg/m^2^ | 42.1 | 42.1 | 0.94 |
| Fat mass, kg | 56.3 | 58.3 | 0.45 |
| Handgrip strength, kg | 33.5 | 31.8 | 0.72 |
| Chair sit to stand, number in 30 seconds | 8 | 8 | 0.97 |
| Six-minute Walk, meters | 371.5 | 360.0 | 0.56 |

Mean data are presented unless otherwise indicated. Statistical comparisons were completed using Mann Whitney U test for continuous and Fishers Exact test for categorical outcomes

*All study participants still met criteria for high adiposity (i.e., obesity) at study end based on waist circumference and measured percent fat mass

*******

******

*****

**Supplemental 3.** Attrition rate details in context of COVID-19 pandemic public health measures

No recruitment was conducted in December 2021, July 2022, or September 2022 due to clinic closures or no eligible consult appointments

*COVID-related public health restrictions were downgraded in July 2021 enabling study recruitment to commence in the fall

**Omicron virus outbreak occurred in January 2022, resulting in increased public health restrictions

***All COVID-related public health restrictions were removed in the province

^◊^Attrition includes participants who withdrew, were removed due to safety concerns, or lost to follow-up

Reference source: Covid timeline in Alberta. <https://calgaryherald.com/news/local-news/two-years-of-covid-19-a-timeline-of-the-pandemic-in-alberta>

**Usual Care**

**(Control)**

**Baseline Assessment**

**POMELO Intervention**

**(12-weeks)**


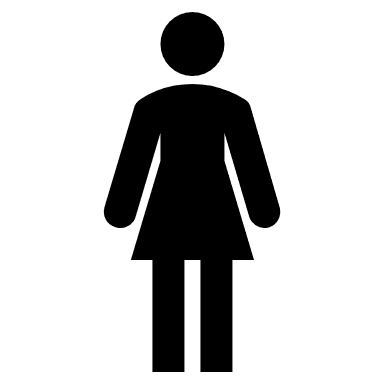

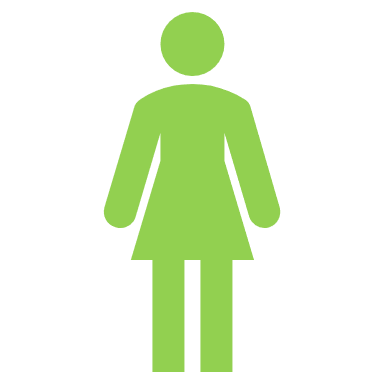

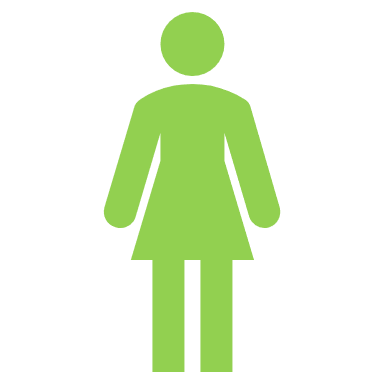

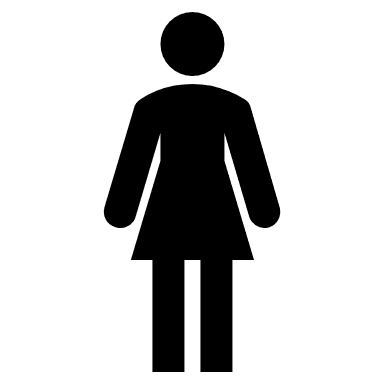

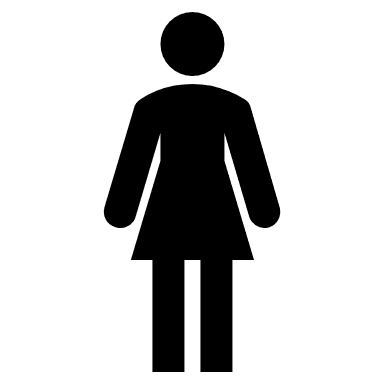

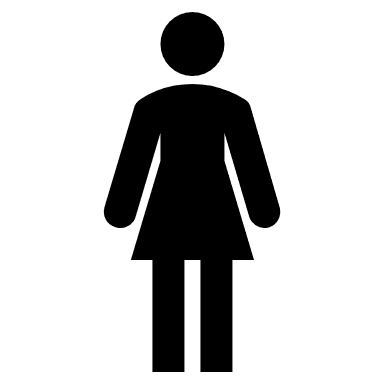

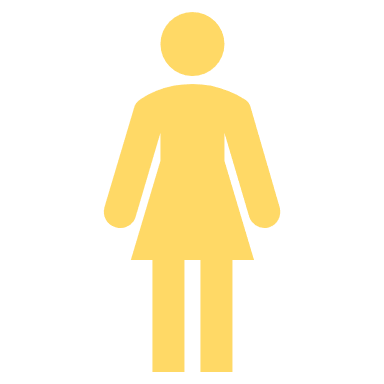

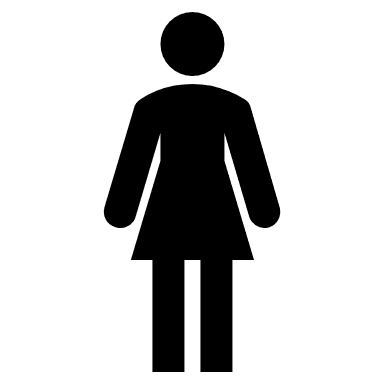

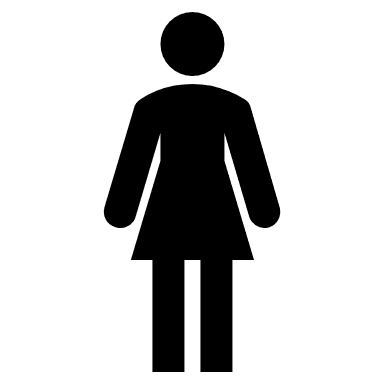

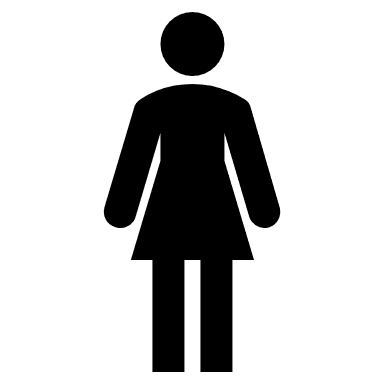

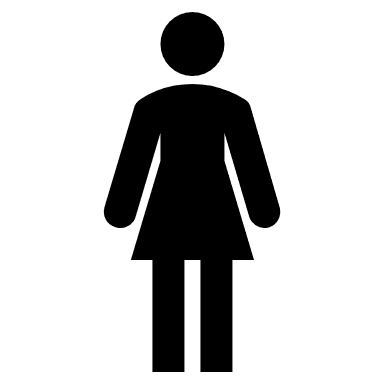

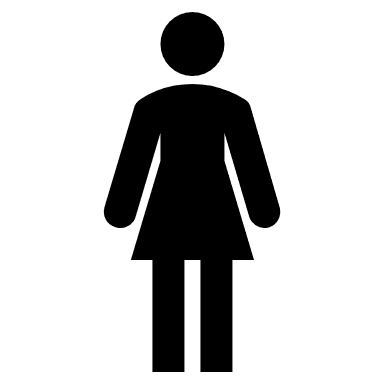

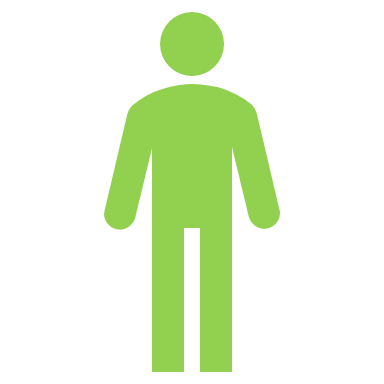

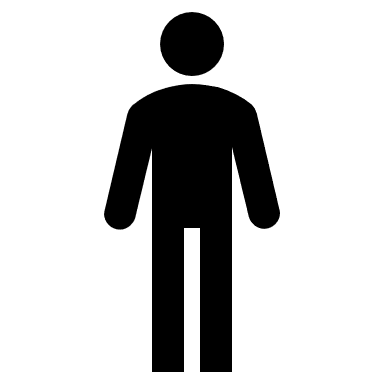

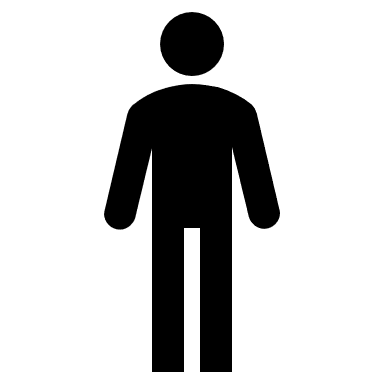

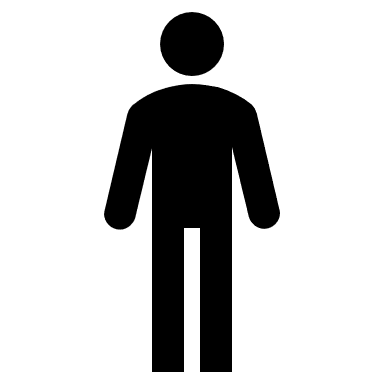

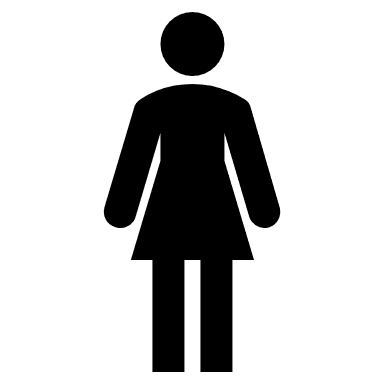

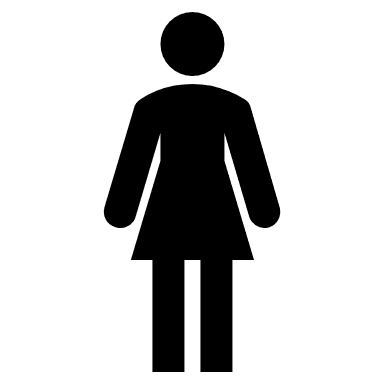

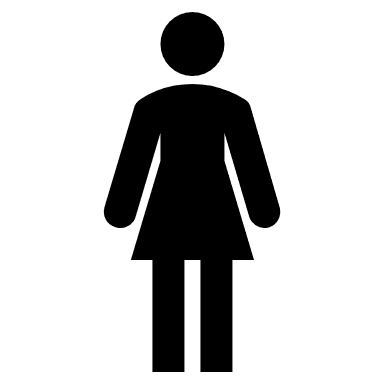

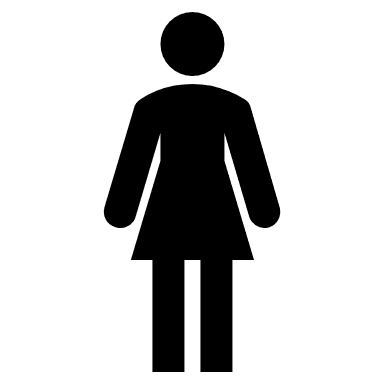

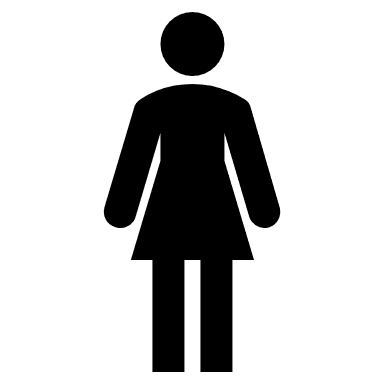

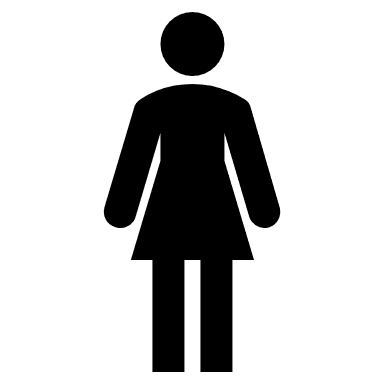

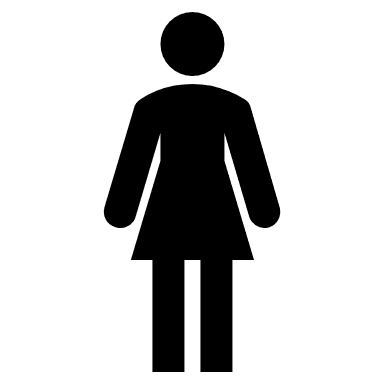

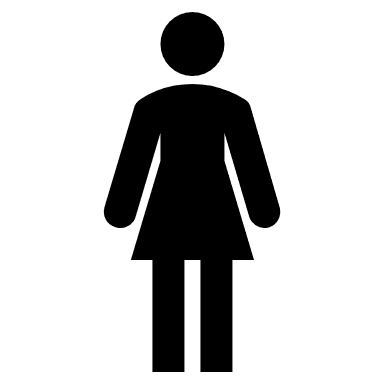

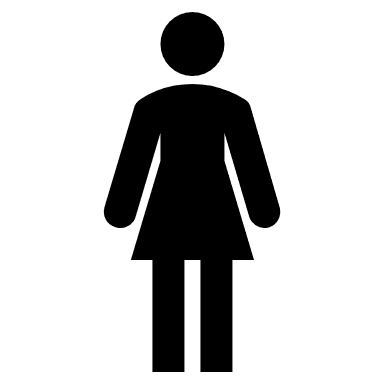

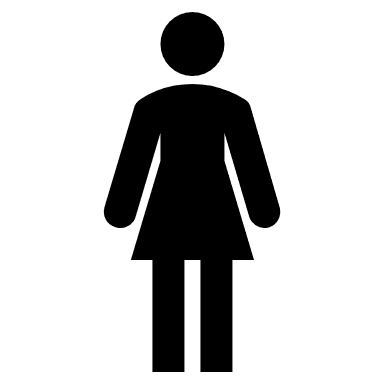

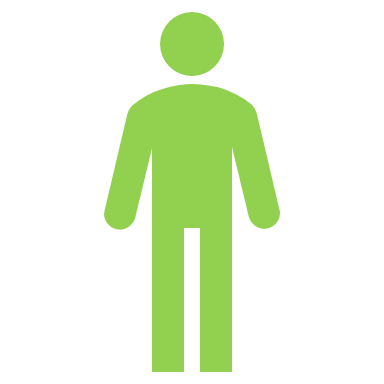

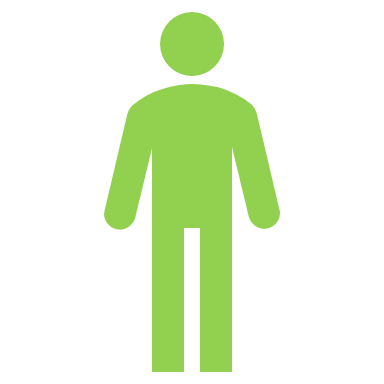

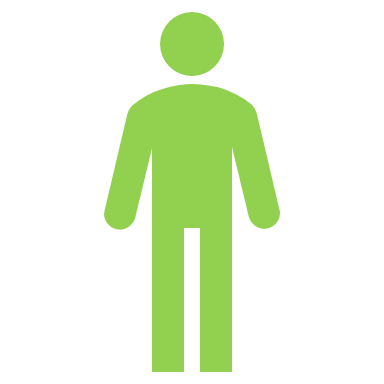

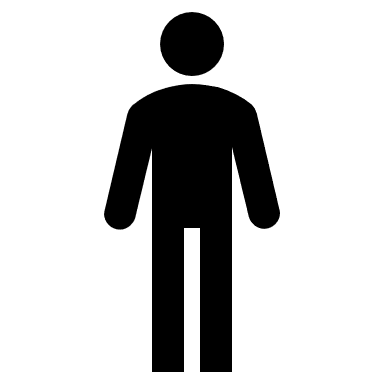

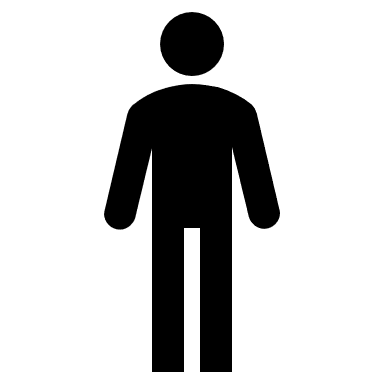

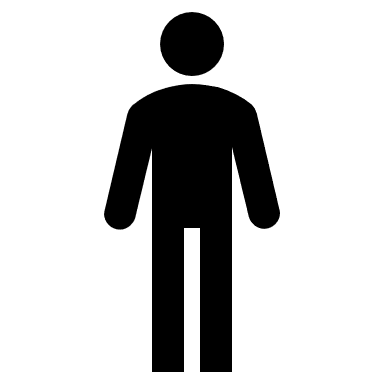

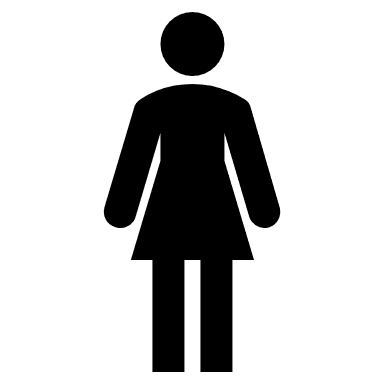

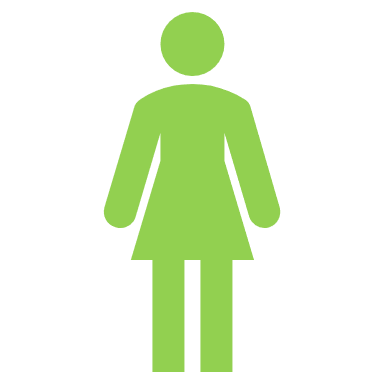

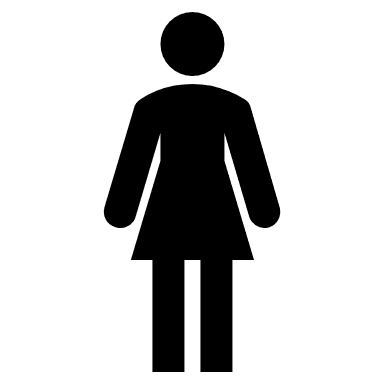

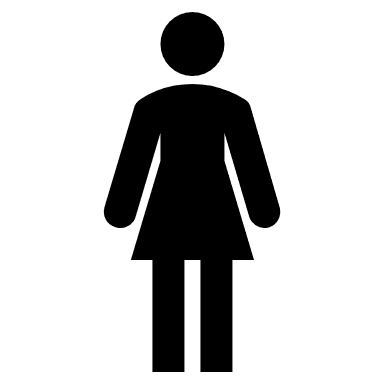

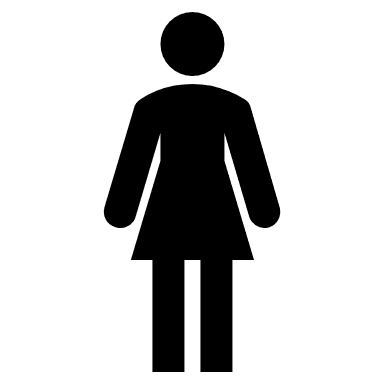

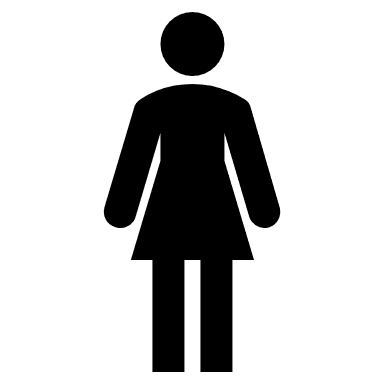

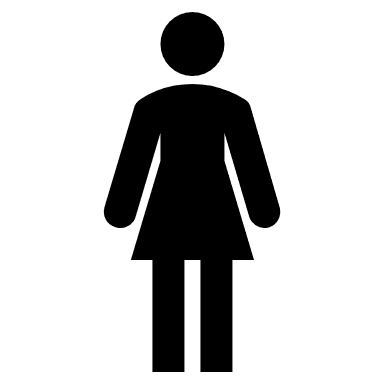

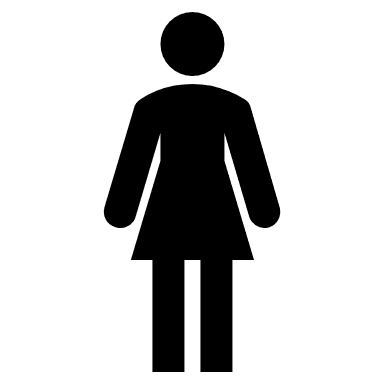

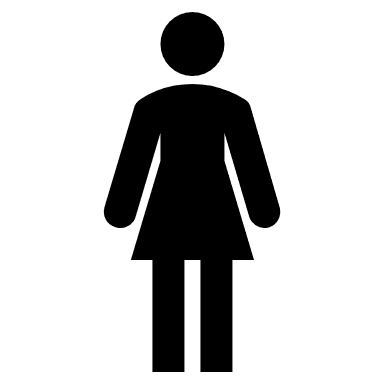

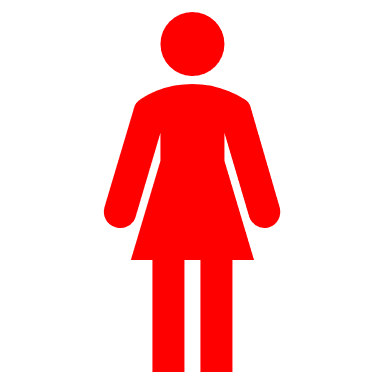

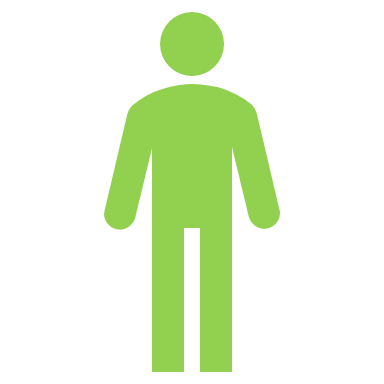

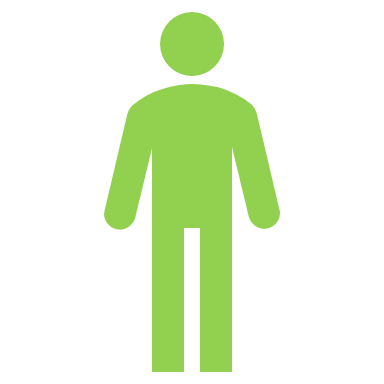

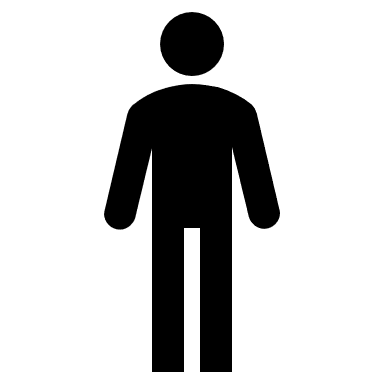

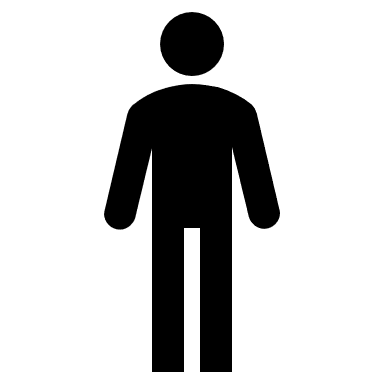

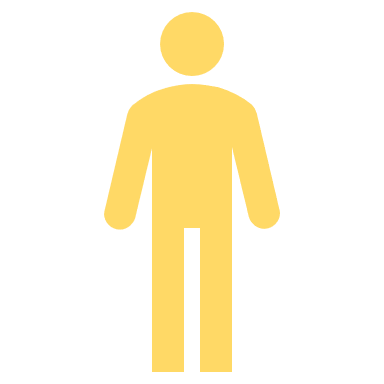

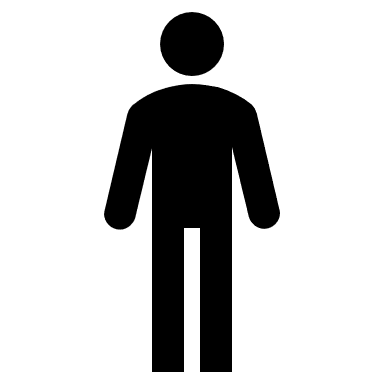

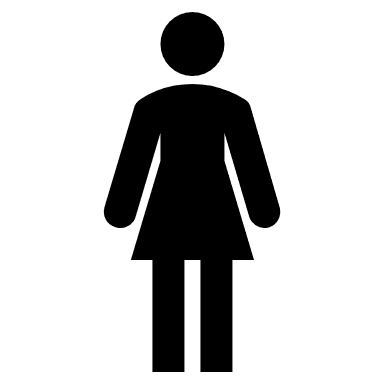

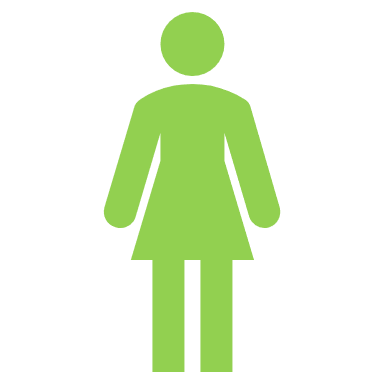

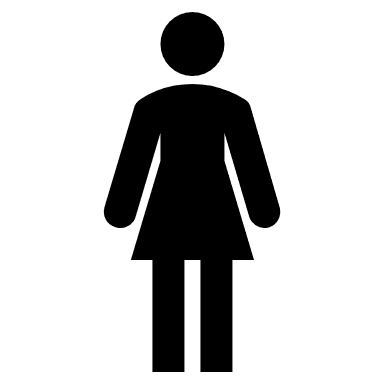

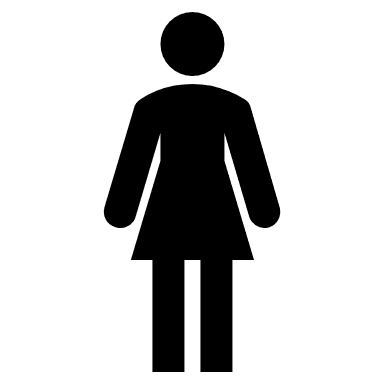

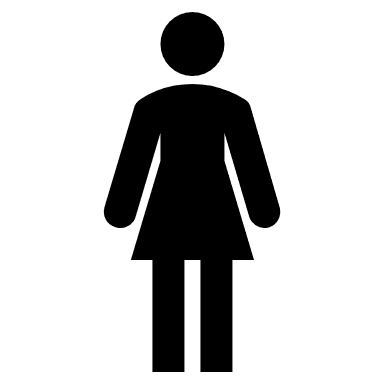

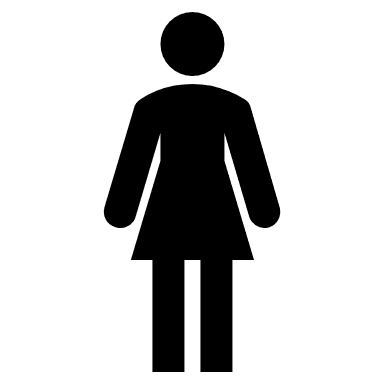

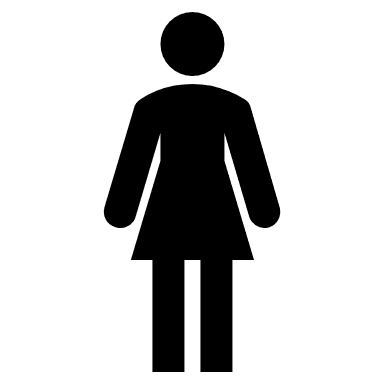

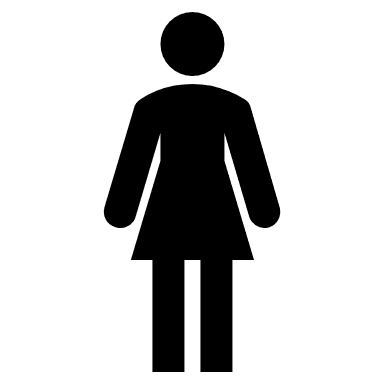

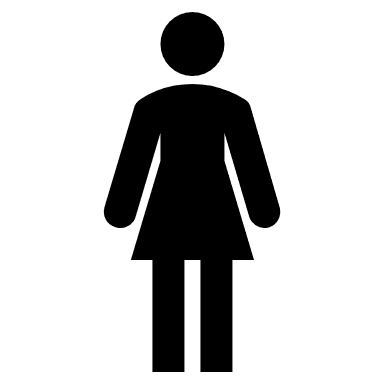

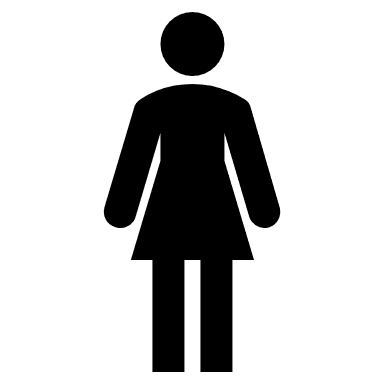

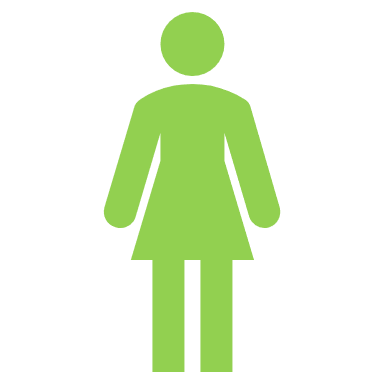

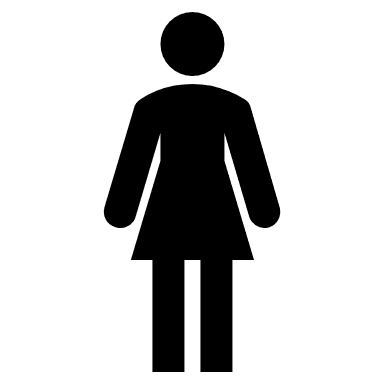

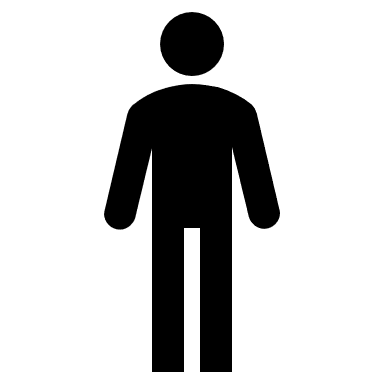

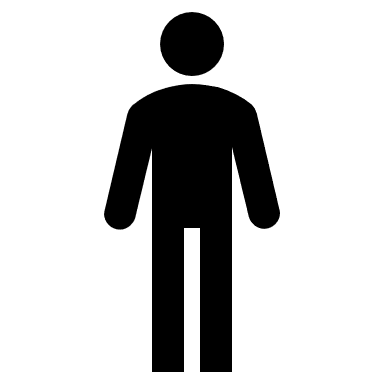

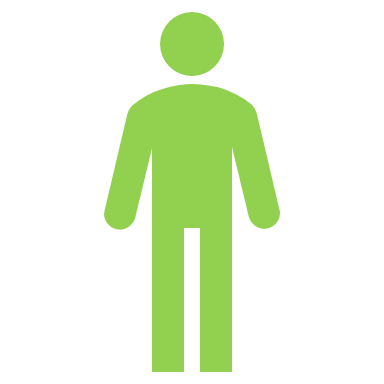

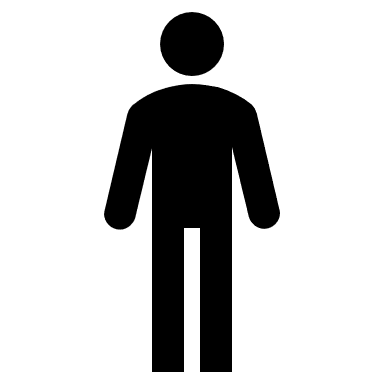


**Unsupported Maintenance Period**

**(6-months)**

**9-Month Assessment**


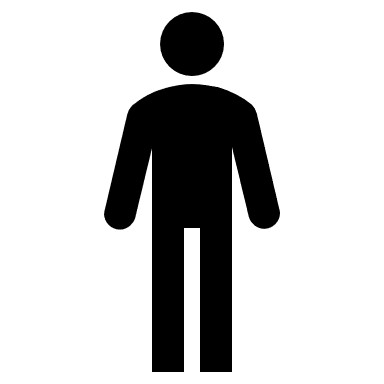

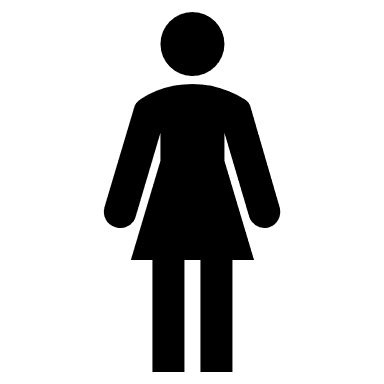


**Legend**

Female

Male

Low Function†

Low Muscle Mass††

Sarcopenic Obesity ‡

**Supplemental 4.** Post-hoc identification of change in sarcopenic obesity status from baseline to the end of the 9-month study period in n=32 study completers (16=POMELO, 16=Usual Care)

† Identified by low absolute maximal handgrip strength, <20 kg in females, and <30 kg in males

†† Identified by low appendicular lean soft tissue/weight, <19.4% in females, and <25.7% in males

‡ Low function + Low muscle mass (+ Obesity*)
